# Assessment of transient ischemic dilation with cardiac MRI

**DOI:** 10.1186/1532-429X-18-S1-P68

**Published:** 2016-01-27

**Authors:** John Lisko, Daniel Morgenstern, Brandon M Mikolich, Nicola B Nicoloff, Ronald J Mikolich

**Affiliations:** 1 grid.261103.70000000404597529Northeast Ohio Medical University (NEOMED), Rootstown, OH USA; 2 Sharon Regional Health System, Sharon, PA USA

## Background

Transient ischemic dilation of the left ventricle may be suggestive of global myocardial ischemia on SPECT stress imaging. While SPECT stress/rest imaging relies on computation-adjusted volumetric data to calculate a stress/rest "TID ratio," measured at 20 to 30 minutes after peak regadenoson stress, cardiac MRI (CMR) allows for the direct measurement of LV wall thickness and cavity dimensions at peak regadenoson stress. This study sought to define a CMR derived normal TID ratio using a cohort of patients with a low probability of coronary atherosclerotic disease (CAD).

## Methods

A standard CMR myocardial perfusion protocol was modified by adding a short axis SSFP imaging sequence at the level of the papillary muscle tips, just before the 1st pass perfusion study at peak regadenoson effect. Parameters for this study included LV end-diastolic diameter (EDD),LV end-systolic diameter (ESD), and myocardial perfusion findings, all of which were assessed at rest and at peak regadenoson stress. Delayed enhancement imaging with gadolinium was also performed in all studies. For this study, TID was defined as an EDD increase with stress. All data was entered into an institutional cardiac imaging database which was then queried for all patients at low risk for CAD, defined by including only those patients with a left ventricle ejection fraction of between 55 and 65%, no evidence of perfusion abnormality, no evidence of previous myocardial infarction or pericarditis by delayed enhanced gadolinium imaging, no more than 1+ valvular heart disease, normal T2 STIR imaging, and no regional wall abnormalities. The ratio of end-diastolic diameter at stress to end-diastolic diameter at rest was calculated for each patient. The upper limit of normal for the TID ratio was assumed to be 2 standard deviations greater than the mean.

## Results

Out of 605 patients that had stress and rest EDD measurements, 63 met the low risk CAD criteria, defined above. The mean EDD at rest was calculated to be 5.50 cm, while the mean EDD at peak stress was calculated to be 5.39, yielding a EDD ratio of 0.98 +/- 0.05. Assuming a normal distribution, the upper limit of normal for the TID ratio would be 1.08.

## Conclusions

CMR allows for the direct measurement of EDD at peak regadenoson stress without the need for computational volume adjustments. This study suggests, in a low risk population, that the upper limit of normal for a TID ratio is 1.08 using CMR. The upper limit of normal TID ratio is 1.2 is by SPECT. These differences may be attributable to variance in the timing of image acquisition and the need for computational volume adjustments with SPECT imaging.

